# Do Antimonite and Silicon Share the Same Root Uptake Pathway by Lsi1 in *Sorghum bicolor* L. Moench?

**DOI:** 10.3390/plants12122368

**Published:** 2023-06-19

**Authors:** Chirappurathu Sukumaran Nair Vidya, Rajpal Shetty, Boris Bokor, Ivana Fialová, Miroslava Luxová, Katarína Jašková, Marek Vaculík

**Affiliations:** 1 Institute of Botany, Plant Science and Biodiversity Centre, Slovak Academy of Sciences, Dúbravská cesta 14, SK-845 23 Bratislava, Slovakia; 2 Department of Plant Physiology, Faculty of Natural Sciences, Comenius University in Bratislava, Mlynska dolina B2, Ilkovicova 6, SK-842 15 Bratislava, Slovakia; 3 Comenius University Science Park, SK-841 04 Bratislava, Slovakia

**Keywords:** antimony (Sb), ascorbate, Lsi1 transporter, malondialdehyde, metalloid uptake pathway, silicon (Si) accumulation

## Abstract

A study was conducted to further develop our understanding of antimony (Sb) uptake in plants. Unlike other metal(loid)s, such as silicon (Si), the mechanisms of Sb uptake are not well understood. However, SbIII is thought to enter the cell via aquaglyceroporins. We investigated if the channel protein Lsi1, which aids in Si uptake, also plays a role in Sb uptake. Seedlings of WT sorghum, with normal silicon accumulation, and its mutant (*sblsi1*), with low silicon accumulation, were grown in Hoagland solution for 22 days in the growth chamber under controlled conditions. Control, Sb (10 mg Sb L^−1^), Si (1mM) and Sb + Si (10 mg Sb L^−1^ + 1 mM Si) were the treatments. After 22 days, root and shoot biomass, the concentration of elements in root and shoot tissues, lipid peroxidation and ascorbate levels, and relative expression of *Lsi1* were determined. When mutant plants were exposed to Sb, they showed almost no toxicity symptoms compared to WT plants, indicating that Sb was not toxic to mutant plants. On the other hand, WT plants had decreased root and shoot biomass, increased MDA content and increased Sb uptake compared to mutant plants. In the presence of Sb, we also found that *SbLsi1* was downregulated in the roots of WT plants. The results of this experiment support the role of Lsi1 in Sb uptake in sorghum plants.

## 1. Introduction

Very little insights are available on the uptake of antimony (Sb) by plants. Antimony is a metalloid belonging to group 15 of the Periodic table along with arsenic (As). Increasing anthropogenic input has made it a source of significant environmental concern. Depending on the degree of mineralization, the changes in the parent material and the various sampling techniques, the concentration of Sb in the Earth’s crust ranges from 0.2 to 0.3 mg Sb kg^−1^ [1,2,3]. Higher levels of Sb were found in the atmosphere, which scientists believe are caused by vehicle emissions, mineral smelting, fossil fuel burning and waste incineration [4]. Despite being non-essential, Sb is taken up by plants and is known to have toxic effects such as stunted development, reduced biomass and photosynthesis, production of reactive oxygen species and lipid peroxidation [5,6,7]. Antimony is largely absorbed by plants through the soil and exists in soil in a variety of oxidation states, including III, V and methylated forms. Among these, SbIII was discovered to be most detrimental to plants [8,9,10]. However, mechanisms of uptake of antimonite (SbIII) and antimonate (SbV) may not be the same. Antimonate (Sb(OH)_6_^−^) transport into the symplasm would require anion transporters of low selectivity and remains unknown [11]. On the other hand, SbIII is a neutral molecule and found to have strong similarity with glycerol in terms of their neutrality, stoichiometry, charge distribution and volume [12,13]. Hence, studies suggest SbIII mimics glycerol and is transported via aquaglyceroporins [12]. Plant aquaporins make up a large and divergent superfamily of major intrinsic proteins (MIPs) and are found in all living organisms [14]. Nodulin26-like intrinsic membrane proteins (NIPs) are a subfamily of MIPs, which are localized to plasma and intracellular membranes and known to mediate the transport of NH_3_, B(OH)_3_ or Si(OH)_3_ [15,16]. Studies showed that Sb transport proteins in plants may belong to NIPs and are expressed in the roots [17].

One of the important and well-characterized members of NIP subfamily is Lsi1, a channel protein mainly expressed in roots that acts as the main silicon (Si) influx transporter [15].

Silicon (Si) is also a metalloid known for its beneficial effects on plants [18]. Many studies have documented the positive role of Si in improving the tolerance against Sb stress. For example, Si decreased the oxidative stress symptoms documented by lower lipid peroxidation, proline accumulation and decreased activity of antioxidative enzymes in Sb stressed roots [6,19]. Plants absorb Si in the form of mono-silicic acid, Si(OH)_4_ by roots either passively or actively [20]. Plants have well-evolved Si transport systems which include Low silicon1 (*Lsi1*), Low silicon2 (*Lsi2*), Low silicon3 (*Lsi3*) and Low silicon6 (*Lsi6*) proteins [21].

Studies showed that plants belonging to Poaceae, Equisetaceae and Cyperaceae accumulate more Si [22]. Sorghum (*Sorghum bicolor* L.), a major food crop belonging to Poaceae family, is known to take up Si in large amounts and form Si-containing aggregates in their roots [23,24]. Antimonite has been shown to be transported by aquaporins of the NIP subfamily, and Si is transported by Lsi1, which is also a member of the NIP subfamily. Therefore, this poses a question whether Lsi1 could participate in Sb uptake in sorghum plants. To answer this question, we conducted Sb uptake and accumulation experiments with sorghum plants with normal silicon accumulation (WT) and its mutant with low Si accumulation lacking Lsi1 (*sblsi1*). The goal of this experiment is to expand our current understanding of Sb uptake in plants.

## 2. Results

### 2.1. Plant Growth

**Figure 1:**
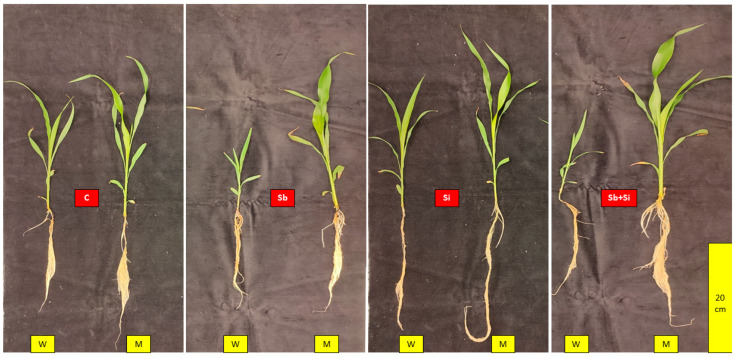
Picture taken 22 DAP (days after planting) depicting the plant growth of the sorghum wild-type (W) and *sblsi1* mutant (M) plants grown under treatments: C (Control), Sb (10 mg Sb L^−1^), Si (1 mM) and Sb + Si (10 mg Sb L^−1^ + 1 mM Si) (Scale bar: 20 cm).

Shoot length of wild type (WT) sorghum plants decreased significantly with the addition of Sb (Figure 1). Meanwhile, Sb had no effect on the shoot length of mutant sorghum plants (Table 1). 

The length of the roots appeared to be reduced in WT plants in the presence of Sb, though the difference was not significant, and root length was found to be unaffected in mutant plants. When mutant plants were compared to control plants, the length and width of the leaves remained unchanged in the presence of Sb, whereas WT plants showed a significant decrease compared to the control. The use of Si in combination with Sb had no effect on any of these parameters (Table 1).

**Figure 2:**
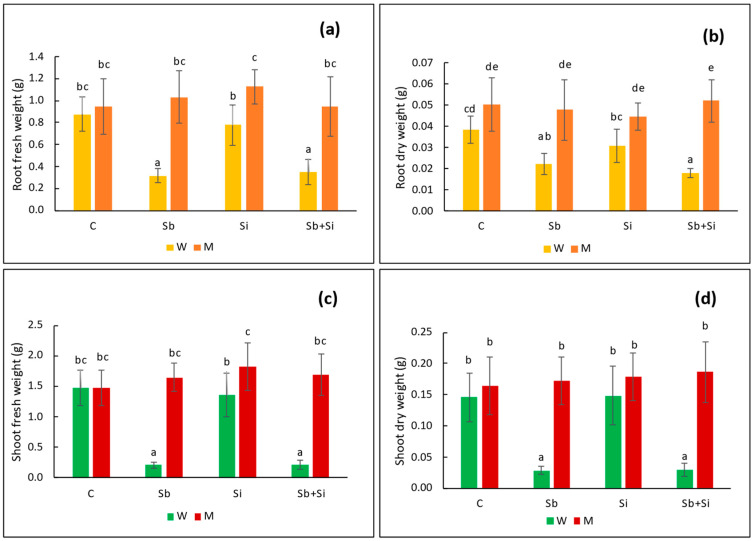
Root fresh weight (**a**), root dry weight (**b**), shoot fresh weight (**c**) and shoot dry weight (**d**) in the sorghum wild-type (W) and *sblsi1* mutant (M) plants grown under treatments: C (Control), Sb (10 mg Sb L^−1^), Si (1 mM) and Sb + Si (10 mg Sb L^−1^ + 1 mM Si). Values are means ± SD (*n* = 3). Different letters indicate significant differences between the treatments at *p* < 0.05.

Under Sb treatment, both shoot fresh and dry biomass decreased dramatically in WT plants (Figure 2c,d). The reduction in fresh weight was more than 85%, and the reduction in dry weight was 80%. The fresh and dry biomass of Si-treated plants was comparable to those of control plants, and the combination of Si and Sb reduced the shoot biomass in the same way that Sb alone did in WT plants. The shoot biomass of mutant plants, on the other hand, appeared unaffected by the presence of Sb, as shown in Figure 2c,d. In the case of root fresh and dry weight, Sb lowered the fresh weight of WT plants by 64% and the dry weight by 42% (Figure 2a,b). There was no difference in fresh and dry root biomass in mutant plants upon the Sb treatment compared to the control. The use of Si had no effect on root biomass of Sb-treated WT and mutant plants.

### 2.2. Lipid Peroxidation

**Figure 3:**
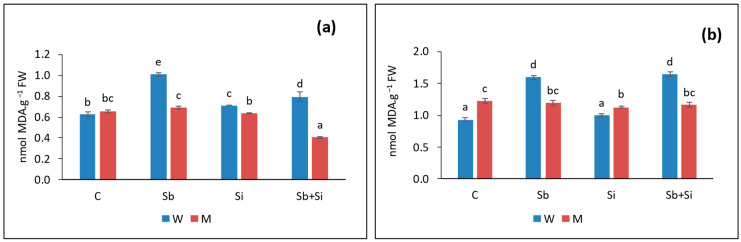
Malondialdehyde (MDA) contents in roots (**a**) and shoots (**b**) in sorghum wild-type (W) and *sblsi1* mutant (M) plants grown under treatments: C (Control), Sb (10 mg Sb L^−1^), Si (1 mM) and Sb + Si (10 mg Sb L^−1^ + 1 mM Si). Values are means ± SD (*n* = 3). Different letters indicate significant differences between the treatments at *p* < 0.05.

Malondialdehyde (MDA) content was used to assess lipid peroxidation levels in roots (Figure 3a) and shoots (Figure 3b). The levels of MDA in both shoots and roots of WT plants grown with Sb were significantly higher compared to the control. Meanwhile, Sb treatments had no effect on MDA content in mutant plants. 

### 2.3. Ascorbate Content

**Figure 4:**
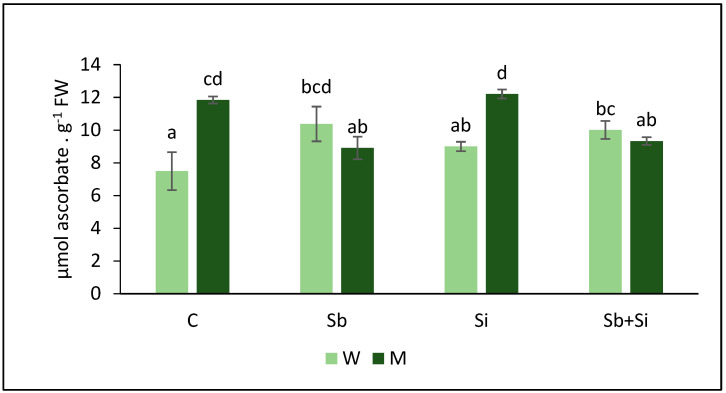
Ascorbate (AsA) content in roots of sorghum wild-type (W) and *sblsi1* mutant (M) plants grown under treatments: C (Control), Sb (10 mg Sb L^−1^), Si (1 mM) and Sb + Si (10 mg Sb L^−1^+ 1 mM Si). Values are means ± SD (*n* = 3). Different letters indicate significant differences between the treatments at *p* < 0.05.

The ascorbate content of mutant plants was discovered to be higher than that of WT plants in the control treatment (Figure 4). When plants were exposed to Sb, the ascorbate content in WT plants increased; however, the opposite was observed in mutant plants. The application of Si alone did not have any impact on ascorbate contents in WT and mutant compared to the control. Additionally, no significant differences were observed between Sb and Sb + Si plants, both in WT and mutant plants. 

### 2.4. Concentration of Sb and Si in Roots and Shoots

**Figure 5:**
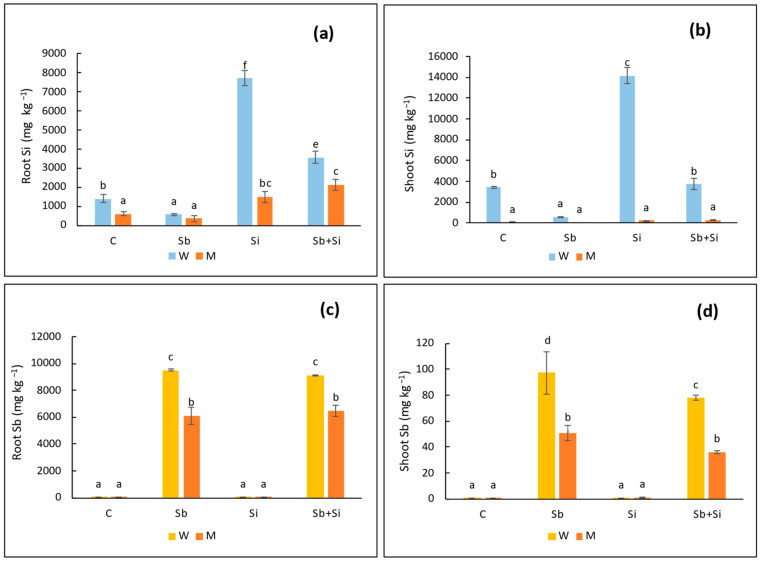
Concentration of Si in roots (**a**) and shoots (**b**); and concentration of Sb in roots (**c**) and shoots (**d**) in sorghum wild-type (W) and *sblsi1* mutant (M) plants grown under treatments: C (Control), Sb (10 mg Sb L^−1^), Si (1 mM) and Sb + Si (10 mg Sb L^−1^ + 1 mM Si). Values are means ± SD (*n* = 3). Different letters indicate significant differences between the treatments at *p* < 0.05.

Concentration of Sb in roots was increased upon the addition of Sb both in WT and mutant plants (Figure 5c). Antimony concentration was higher in roots than in shoots, and Sb concentration in mutant plants was nearly a two-fold decrease when compared to WT plants. The root Sb concentration was nearly identical in the Sb + Si treatment versus Sb alone in roots of both WT and mutant plants. The same results were observed in Sb concentration in shoots, where shoot Sb concentration in mutant plants was reduced about 50% compared to WT (Figure 5d). When Sb and Si were exposed together, there was a significant reduction in Sb concentration in shoots of WT plants when compared to Sb treatment alone. However, the shoot Sb concentration was unaltered in Sb and Sb + Si in the case of mutant plants. 

The addition of Si to the growth media increased the Si concentration in both roots and shoots (Figure 5a,b). In WT plants, the concentration of Si in shoots was greater than in roots, while the opposite trend was observed in mutant plants, where Si was comparatively less in shoots than roots. At an external concentration of 1 mM Si, the translocation factor for Si in WT plants was determined to be 1.83, and when Si was combined with Sb, it was reduced to one. In WT plants, Sb + Si treatment significantly reduced Si concentrations in both roots and shoots. Mutant plants had a much lower Si concentration (five times lower) in roots than WT plants. Moreover, compared to WT, Si concentration in mutant shoots was 60 times lower. The accumulation of silicon in the aerial parts of mutant plants was discovered to be negligible.

### 2.5. Expression of SbLsi1 Gene in Roots 

**Figure 6:**
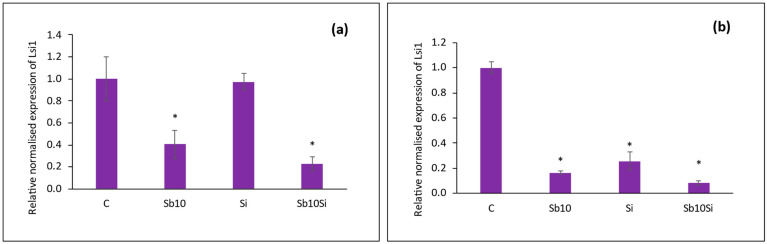
Relative normalized expression of *SbLsi1* in roots of wild-type sorghum plants (**a**) 5 DAP and (**b**) 22 DAP under treatments: C (Control), Sb (10 mg Sb L^−1^), Si (1 mM) and Sb + Si (10 mg Sb L^−1^ + 1 mM Si). Gene expression for the control was set as 1.0. Statistically significant differences between control and treated plants were analyzed via Student’s *t* test (*p* < 0.05) and are denoted as *. Values are means ± standard deviation. The mean values are based on three technical and three biological replicates.

In mutant plants, the *SbLsi1* gene was not expressed. The expression of *SbLsi1* in WT plants changed throughout time. *SbLsi1* expression was comparable to the control at 5 DAP in Si treatment (Figure 6a), but it decreased by a factor of four times with Si addition at 22 DAP (Figure 6b). *SbLsi1* expression was downregulated in Sb-treated roots of plants taken at 5 and 22 DAP, with the latter showing a greater reduction.

## 3. Discussion

We investigated the sorghum Lsi1 (*sblsi1*) mutant and its WT plants grown under SbIII exposure in this experiment. Studies showed that *sblsi1* plants have low silicification levels, implying that Lsi1 is the main gateway for Si absorption in sorghum plants [25], which is consistent with our findings of very low silicon uptake by the mutant plants. As per the study conducted by Markovich et al. (2022) [26], wild sorghum and its corresponding *sblsi1* mutant had no structural differences: they were upright, similar in height, and had identical root structure except mutant plants, which lack the Lsi1 channel in roots.

Even though antimony is a non-essential element with no role in plant metabolism, it is taken up by plants. It has been reported that concentrations of 5 to 10 mg Sb kg^−1^ in plants are potentially toxic and can disrupt normal plant function [27]. In our study, Sb at 10 mg Sb kg^−1^ was found to decrease the fresh and dry biomass of root and shoot as well as the shoot length, leaf length and width of WT sorghum plants. The severity of toxicity is related on the plant type, dose and Sb species [28]. Decreased root and shoot biomass is considered as the consequence of increased uptake of Sb [29,30,31]. On the other hand, Sb did not affect the growth and biomass of mutant plants. Many studies have revealed that Si helps to improve plant tolerance against metal(loid) stress, such as cadmium (Cd) [32,33], chromium (Cr) [34], As [35] and others. This may not be true in all cases, as we found that the application of Si did not improve the biomass of Sb-treated WT sorghum plants in our study, which, moreover, agrees with the findings of Masarovič et al. (2012) [36], who found that Si had no positive effect on sorghum biomass production either alone or in combination with Zn.

The peroxidation of cell membrane lipids (LPO) Is considered as one of the most damaging processes occurring in every living organism. The reactive oxygen species (ROS) generated due to any stress enhance the extent of LPO, which results in the loss of membrane integrity [37]. The presence of MDA and thiobarbituric acid reactive substances (TBARS) is considered as an indicator of oxidative stress and lipid peroxidation in plants. Several studies showed an increased MDA content with increasing concentration of Sb in various plants. For example, Vaculíková et al. (2014) [6] observed increased levels of MDA in roots of *Zea mays* upon Sb addition. Similarly, in our study, the MDA contents were significantly higher both in roots and shoots of Sb-treated WT plants compared to the control, indicating an enhanced oxidative stress due to Sb uptake which in turn reduced the growth of plants. On the other hand, MDA levels in mutant were same as that of the control, showing there was no oxidative stress due to Sb treatment. Ascorbate (AsA) is an important component of plant antioxidant system. Plants tend to produce more AsA for coping with stress including metals and metalloids, as explained by Ortega et al. (2017) [7]. When Sb was applied to WT plants, we found that the level of AsA in the roots increased, and there was no change in the shoots. Sb accumulation was found to be higher in roots than in shoots of WT sorghum plants, implying a larger antioxidant response by roots to Sb. These data suggest that treating WT plants with 10 mg Sb kg^−1^ caused severe stress and stunted growth, in contrast to sblsi1 mutants that did not appear to be affected by Sb.

The addition of 1 mM Si to the growth media resulted in higher Si levels in the roots and shoots of WT sorghum plants. Gramineae plants, such as sorghum, absorb a lot of Si [18,38], which explains why WT plants have a lot of Si in their shoots. Despite the absence of the Lsi1 transporter in mutant plants, low levels of Si were discovered in their roots, which could be due to passive apoplasmic transport of Si, or possibly, it might also have resulted from residual root surface Si content. In contrast to WT plants, higher Si levels were also found in roots than in shoots in Si-mutant rice plants [39]. This may be due to the fact that root tissues need some Si, which may be taken up through apoplasmic space [25]. Because mutant plants lack Lsi1, the main cellular Si uptake pathway, Si in roots of mutant plants cannot be transported symplasmically, and as a result, Si does not accumulate in the shoots of these plants. We discovered that sorghum mutant plants lacking the silicon transporter Lsi1 are more resistant to Sb. With Sb uptake reduced by 50% relative to WT, we suppose that Si and Sb may share the same transport mechanism. Similar resistance was shown in case of germanium (Ge), a metalloid that was later utilized as a selection parameter to isolate mutants in rice that were deficient in Si absorption [40]. We observed a negative interaction between Si and Sb in WT, with Si absorption being reduced by 50% in the presence of Sb in roots and shoots, while Sb concentration remained constant in roots but decreased in shoots. This contrasts with studies that found Si inhibited absorption of Sb [41], As [42], Se [43] and Cd [32]. Reduced Si concentration in Sb + Si treatment of WT could be due to Sb and Si competing for the same absorption site, or Sb was discovered to be very toxic to WT plants with increased MDA content in roots and decreased root biomass, causing oxidative damage to the root and eventually reducing Si uptake. Similarly, toxic elements have been reported to prevent the uptake of other essential elements. For example, the treatment of sunflower plants with Sb reduced the uptake of Mg, Fe, Cu and Zn, which corresponds with high oxidative stress in roots [7]. 

The *Lsi1* gene was not expressed in roots of mutant plants in our study, which explains the low Si uptake compared to WT plants. The present study showed that when 1 mM Si was supplied, the *Lsi1* expression was downregulated after 22 days of planting with or without Sb stress. Similar results were recorded by Ma et al. (2006) [44], where the expression of *Lsi1* in *Oryza sativa* reduced by one-fourth when Si was provided in the nutrient solution. However, it contradicted studies that suggested otherwise. For example, the expression of *Lsi1* was found to be upregulated and resulted in an increased uptake of Si in rice plants with or without the presence of Cd [45]. Enhanced expression of a transporter gene helps in metal(loid) accumulation [46]. However, in our study, the downregulation of *Lsi1* was observed in Sb-treated roots with greater accumulation of Sb in WT plants. Meanwhile mutant plants had taken up significantly lower amounts of Sb. When Sb and Si were applied together, the expression of *Lsi1* was further reduced, and thus, the uptake of Si was reduced as well. This could be due to a plant’s response to stress to reduce the further uptake of Sb.

In conclusion, wild-type sorghum plants showed increased root and shoot Sb accumulation compared to their respective *sblsi1* mutant plants, lacking the expression of Lsi1, a key protein responsible for Si uptake by plant roots. Additionally, WT plants showed higher oxidative stress and lower biomass when compared to *sblsi1* mutant plants. Mutant plants lacking the silicon Lsi1 transporter in their roots appear to be more resistant to Sb uptake and its associated toxicity. This study found a negative interaction between Sb and Si uptake, with Sb-induced downregulation of Lsi1 indicating that Sb and Si may use similar absorption mechanisms. However, mutant plants still accumulated a certain portion of Sb in their roots and shoots. Therefore, we suggest that Lsi1 may not be the only transporter involved in the uptake of Sb, but it plays a significant role in this process. 

## 4. Materials and Methods

### 4.1. Experimental Set Up

Seeds of wild-type sorghum (*Sorghum bicolor* (L.) Moench, line BTx623) with normal silicon accumulation (WT) and its low-silica mutant (*sblsi1*) (knockout mutant) [25] were obtained from The Smith Institute of Plant Sciences and Genetics in Agriculture, the Hebrew University of Jerusalem, Israel. Seeds were sterilized for 10 min by soaking them in a solution of 3% sodium hypochlorite, followed by several thorough washes with distilled water and a two-hour soak in water. These seeds were subsequently allowed to germinate for 72 h at 26 °C in the dark on moist tissue paper. To determine moderate toxic concentration of Sb, germinated seedlings were transferred to 1L pots with Hoagland solution (pH 6) [47] with different concentrations of Sb (0, 5, 10, 20 and 40 mg Sb L^−1^). Solutions were changed every other day. Plants were cultivated for 10 days. We measured the plant growth characteristics such as plant height, leaf length and width, fresh weight and dry weight of root and shoot. From the results, we found that Sb over the dose of 10 mg L^−1^ was highly toxic, and plants barely survived. On the other hand, 5 mg Sb L^−1^ was slightly toxic to plants. Hence, we selected 10 mg Sb L^−1^, which we found to be moderately toxic to the sorghum plants for further experiments. In the main experiment, 3-day-old, germinated seedlings were transferred to 5.5 L pots with Hoagland solution with or without Sb and with or without Si 1mM. Antimony was applied in the form of potassium antimony tartrate hemihydrate (SbIII) and Si in the form of sodium silicate solution (Sigma). Plants were grown for 22 days in a growth chamber at a temperature of 25 °C, 16 h photoperiod, and 200 μmol m^−2^ s^−1^ photosynthetically active radiation (PAR). At least three replicates from each treatment were evaluated. 

Four treatments were applied:

1. Control (C): Hoagland solution without Sb and Si.
2. Antimony (Sb): Hoagland solution with 10 mg Sb L^−1^.
3. Silicon (Si): Hoagland solution with 1 mM Si.
4. Antimony + silicon (Sb + Si): Hoagland solution with 10 mg Sb L^−1^ and 1 mM Si.

### 4.2. Determination of Growth Parameters

After 22 days, the plants were removed from the solution, rinsed with tap water and distilled water, and blotted with tissue paper. Plants were then divided into below- and above-ground portions. Shoot length and seminal root length were measured, as well as the length and width of the third fully developed leaf. Roots and shoots were weighed immediately and recorded as fresh weight. The biomass was then dried in an electric oven at 60°C for three days, and the residual weight was recorded as dry weight.

### 4.3. Determination of Sb and Si Concentrations in Roots and Shoots

Dried roots and shoots were ground to fine powder and double-step digested in HNO_3_ and H_2_O_2_; for Si analysis, HF was also applied. The concentration of Sb and Si was evaluated by inductively coupled plasma mass spectrometry (ICP) in the Geoanalytical Laboratories of the Institute of Geomaterials, Faculty of Natural Sciences, Comenius University in Bratislava, Slovakia.

Translocation factor was calculated as follows:

TF = (metalloid)shoot/(metalloid)root

### 4.4. Determination of Lipid Peroxidation 

The level of membrane lipid peroxidation was measured using thiobarbituric acid (TBA) and expressed as malondialdehyde (MDA) content [48]. The samples were homogenized with 2.5 mL trichloroacetic acid before being centrifuged at 12000 rpm for 10 min. The clean sample was then mixed with 2 mL of TCA + TBA solution and boiled for 30 min at 95 °C. It was then rapidly cooled in an ice bath to about 10 °C. The MDA absorption in reactive mixture was recorded spectrophoto-metrically at 532 nm after subtracting the non-specific absorption at 600 nm, and extinction coefficient (ε = 155 mM^−1^ cm^−1^) has been used for calculation of MDA concentration. 

### 4.5. Ascorbate Assay

The Fe^3+^-to-Fe^2+^ reduction in an acidic environment is the basis for the ascorbate (AsA) assay. Fe^2+^ forms a chelate compound with bathophenanthroline, resulting in a darker color absorbing at 534 nm [49]. The ascorbate assay reaction mixture was mixed with 0.1 mL of sample, 0.15 mL of 20% TCA and 0.65 mL of absolute ethanol, and then it was incubated for 10 min at room temperature; then, 0.15 mL of 0.24% N-ethylmaleimide in ethanol and 0.15 mL of 20% TCA were added. The color was produced by mixing 1.5 mL of ethanol with 0.15 mL of 0.03% FeCl_3_, 0.15 mL of 0.5% bathophenanthroline and 0.15 mL of 0.4% H_3_PO_4_, before incubating the mixture for 90 min at 30 °C. The concentrations were determined using standard curves for AsA.

### 4.6. RNA Extraction and Real-Time PCR Analyses

From RNA extraction to real-time PCR, all the experiments were carried out using commercially available kits in accordance with the manufacturer’s instructions. RNA was extracted from frozen root samples that were taken 5 and 22 days after planting (at a distance of 2 cm from the apex). Using the Spectrum Plant Total RNA kit (Merck), total RNA was extracted, and DNase I was treated as directed, except for the DNase I treatment time being increased to 60 to 70 min. By using a NanoDropTM 1000 spectrophotometer (Thermo Fisher Scientific, Erlangen, Germany), the concentration of RNA and sample purity were determined, and RNA integrity was examined using agarose (1%) gel electrophoresis. The synthesis of first strand of cDNA was performed by iScript™ cDNA Synthesis kit (BIO-RAD). Afterwards, the expression level of *SbLsi1* gene was performed by real-time PCR method. Specific cDNA was amplified by Luna^®^ Universal qPCR Master Mix (New England Biolabs, Ipswich, MA, USA) with the set of primers listed in Soukup et al. (2017) [23]. The *serine/threonine-protein phosphatase PP2A-1 catalytic subunit* (*PP2A*, NCBI reference: XM_002453490.2; left 5′ CACAGTCTTCAGTGCGCCTA 3′ and right 5′ AGTAGTCTGGGGTTTTGCGG 3′) was used as a reference control (internal control of expression level) and its stability was evaluated according to Livak and Schmittgen (2001) [50]. Apart from *PP2A*, *actin* (NCBI reference: XM_002456645.1; Soukup et al. 2017 [23]) and *eukaryotic initiation factor 4A* (*EIF4A*, NCBI reference: XM_002451491.2; left 5′ TGGACTGGCTCACAGACAAG 3′ and right 5′ CAAGTGAGACCTGCTGGACA 3′) were also assessed for the stability as reference genes; however, the analysis excluded these two genes. Real-time PCR reactions were performed in 96-well plates on Light Cycler II 480 (Roche) and melt curve analysis of amplification products was included at the end of each run of the qPCR reaction. Relative changes in gene expression were estimated according to Pfaffl (2001) [51], and Student’s *t* test was used for statistical analysis (Statgraphics centurion 15.2.05). 

### 4.7. Statistical Analysis

For all the statistical calculations, the SPSS software (IBM Corp. Released 2013. IBM SPSS Statistics for Windows, Version 21.0. Armonk, NY: IBM Corp) and Excel (Microsoft Office 2017) were used. For comparison of mean difference between treatments, data from the studies were subjected to one-way ANOVA followed by Tukey’s post hoc test. Each treatment was evaluated with at least three biological and three technical replicates. A *p* value < 0.05 was defined as significant.

## Figures and Tables

**Table 1:** Root length, shoot length, leaf length and width of the sorghum wild-type (W) and *sblsi1* mutant (M) plants grown under treatments: C (Control), Sb (10 mg Sb L^−1^), Si (1 mM) and Sb + Si (10 mg Sb L^−1^ + 1 mM Si). Values means ± SD (*n* = 10). Different letters indicate significant differences between the treatments at *p* < 0.05.

*Treatments*		*Root Length* *(cm)*	*Shoot Length* *(cm)*	*Leaf Length* *(cm)*	*Leaf Width* *(cm)*
***C***	W M	23.5 ± 4.4 abc 28.95 ± 3.5 cd	39.03 ± 1.1 bc 40.95 ± 2.2 bc	12.37 ± 0.8 c 12.4 ± 0.46 c	0.9 ± 0.06 b 0.91 ± 0.08 b
***Sb***	W M	19.81 ± 3.9 a 27.17 ± 2.8 cd	19.92 ± 2.6 a 41.91 ± 1.8 bc	4.8 ± 0.26 a 12.52 ± 0.57 c	0.65 ± 0.05 a 0.93 ± 0.08 b
***Si***	W M	26.1 ± 5.6 bc 32.56 ± 5.8 d	38.3 ± 3.5 b 42.7 ± 2.5 c	12.13 ± 0.55 c 12.02 ± 0.50 c	0.94 ± 0.1 b 0.91 ± 0.07 b
***Sb + Si***	W M	20.53 ± 2.4 ab 25.2 ± 4 abc	21.01 ± 2.9 a 42.06 ± 4.1 bc	5.78 ± 0.48 b 12.73 ± 0.61 c	0.63 ± 0.04 a 0.99 ± 0.07 b

## Data Availability

All data generated or analyzed during this study are included in this published article.
